# Anthocyanidins-enriched bilberry extracts inhibit 3T3-L1 adipocyte differentiation via the insulin pathway

**DOI:** 10.1186/1743-7075-8-14

**Published:** 2011-03-08

**Authors:** Rieko Suzuki, Masami Tanaka, Masakatsu Takanashi, Aashiq Hussain, Bo Yuan, Hiroo Toyoda, Masahiko Kuroda

**Affiliations:** 1 Department of Molecular Pathology, Tokyo Medical University, Tokyo, Japan; 2 Department of Clinical Molecular Gene, Tokyo University of Pharmacy and Life Science, Tokyo, Japan

## Abstract

**Background:**

Obesity and metabolic syndrome are important public concerns, and there is increasing demand for effective therapeutic strategies. Flavonoids are expected to improve the risk factors associated with metabolic syndrome. Anthocyanidins are a kind of flavonoids; well known for their anti-oxidative, anti-inflammatory and anti-tumor properties. However, their effects on adipocytes and molecular systems are not well defined. In this study, we examined the effects of anthocyanidins-enriched bilberry extracts on adipocyte differentiation.

**Methods:**

Utilizing 3T3-L1 cell line, we investigated that bilberry extracts and anthocyanidins induced inhibition of lipid accumulation during adipogenesis. To identify what is the most important bilberry mediated-effect, we analyzed the expressions of key transcriptional factors associated with adipocyte differentiation by Real Time (RT)-PCR. From the results of RT-PCR, we hypothesized that bilberry extracts and anthocyanidins blocks insulin signal, we determined the phosphorylation of tyrosine residues of insulin receptor substrate 1 (IRS1) protein by western blotting analysis. In addition, we compared the whole-genome expression profiles of early stage of adipocyte differentiation under four different growth conditions (DMSO, bilberry, two anthocyanidins) by microarray analyses and Gene Set Enrichment Analysis (GSEA).

**Results:**

Exposure to bilberry extracts and anthocyanidins during adipocyte differentiation inhibited 3T3-L1 differentiation. During this period, bilberry extracts and anthocyanidin significantly decreased a key adipocyte differentiation-associated marker, peroxisome proliferator-activated receptor- *γ *(Ppar *γ *) and sterol regulatory element-binding protein 1c (Srebp1c). Western blotting analysis showed that bilberry extracts and anthocyanidin decreased the phosphorylation of tyrosine residues of IRS1. In addition, microarray experiments and GSEA data revealed significantly altered expression of the known genes of the insulin pathway in cells treated with bilberry extracts or anthocyanidins in the early differentiation stages.

**Conclusions:**

Our data demonstrate that anthocyanidin enriched bilberry extracts strongly inhibit the adipocyte differentiation via the insulin pathway. Furthermore, bilberry extracts might be used as a potential complementary treatment for the obese patients with metabolic syndrome.

## Background

Obesity is the main cause of metabolic syndrome. It can lead to various complications, including hardening of the arteries and an increased risk of cardiovascular diseases, and therefore has a large impact on healthcare in both developed and developing countries [1]. The development of obesity is characterized by both increased adipocyte number (hyperplasia) and size (hypertrophy) [2], which is regulated by genetic, endocrine, metabolic, neurological, and nutritional factors [3,4]. Accordingly, understanding the mechanisms by which particular nutrients affect adipocyte differentiation would help prevent obesity and its associated diseases.

A variety of naturally occurring flavonoids have been of recent interest because accumulating evidence indicates that these compounds have beneficial health effects and are relatively safer than drugs [5,6]. Bilberry contains rich flavonoids [7], specifically anthocyanidins. Anthocyanidins have a wide range of biological functions and health benefits, such as anti-oxidative, anti-inflammatory and anti-tumor properties [8,9]. Interestingly, when rats fed on hyperlipidemic diets supplemented with bilberry extracts, their plasma triglyceride levels decreased in proportion with the amount of bilberry extracts [10]. Also, anthocyanin (glycoside of anthocyanidin)-rich diets from purple corn or Moro orange have been shown to ameliorate obesity and insulin resistance in mice fed on a high-fat diet [11-13]. However, the molecular mechanisms underlying these properties remain unknown and require further investigation in cells.

Adipocyte differentiation requires the synergistic action of multiple adipogenic transcription factors [14]. The first indication that this transcriptional network is activated is the involvement of CCAAT/enhancer-binding protein (C/ebp) family. C/ebp *β *and C/ebp *δ *are expressed in the early phase of differentiation, and they subsequently induce transcription of Ppar *γ *and C/ebp *α *[15]. Ppar *γ *and C/ebp *α *are two master regulators of adipocyte differentiation. They slog cooperatively to direct the final phase of adipocyte differentiation by activating the expression of adipocyte-specific genes, such as fatty acid synthetase, fatty acid-binding protein, leptin and adiponectin [16]. Insulin-regulated GLUT4 glucose transporters embedded in granules are transferred and merged to cell-face membrane and increase cellular glucose-uptake. As a result, lipid accumulation proceeds and adipocyte gets lipid droplet [14]. Recent investigations suggest that the lag between the appearance of C/ebp *β *and Ppar *γ *expression is due to the time required to synthesize the additional proteins that facilitate C/ebp *β *activity [16]. Specifically, the transcription of Kruppel-like factor (Klf5) is activated by C/ebp *β *and C/ebp *δ *, and Klf5 in concert with these C/ebps contributes to the induction of Ppar *γ *[17]. It is likely that additional factors in parallel pathways are induced early and converge on Ppar *γ *downstream of C/ebp *β *and C/ebp *δ *, such as Srebp1c transcription factor. Srebp1c may regulate adipocyte differentiation by increasing the transcriptional activity of Ppar *γ *, and Srebp1c is expression is significantly enhanced in response to insulin [18,19]. It has already been reported that the transcriptional network of adipocyte differentiation is dominated by three pathways (insulin, glucocorticoid and cAMP) [16].

This study was designed to evaluate the anti-adipogenic action of the bilberry extracts and anthocyanidins during differentiation of 3T3-L1 cells. Specifically, we aimed to identify the molecular mechanisms underlying the effects of bilberry extracts and anthocyanidins on the transcriptional network and explore the major pathways involved with their inhibitory effects.

## Methods

### Chemical reagents

Bilberry extracts (containing 25% anthocyanidins) were purchased from (Tokiwa Phito Chemical, Chiba, Japan). Anthocyanidins (pelargonidin, cyanidin, delphinidin, peonidin, and malvidin) were purchased from Funakoshi Co., Ltd. (Tokyo, Japan). Since bilberry extracts and all anthocyanidins are oil soluble, hence they were dissolved in DMSO, and stored in the dark at 4°C and the measured pH was 8.0 approximately. For each experiment, cells received bilberry extracts or anthocyanidins premixed with the culture medium. The final concentration of DMSO in the medium was 0.1% throughout the experiments.

### Cell culture and adipocyte differentiation

3T3-L1 preadipocytes (American Type Culture Collection, Manassas, VA) were grown to confluence in DMEM (Sigma-Aldrich, Tokyo, Japan) supplemented with 10% fetal calf serum and penicillin (100 U/ml)/streptomycin (100 *μ*g/ml). Adipocyte differentiation was induced using an Adipogenesis Assay Kit (Chemicon International, Temecula, CA). On day 0, the cells were induced with initiation media (10 *μ*g/ml insulin, 1 *μ*M dexamethasone and 0.5 *μ*M IBMX in DMEM supplemented with 10% fetal calf serum). On day 3, the initiation media was replaced with progression media (10 *μ*g/ml insulin in DMEM supplemented with 10% fetal calf serum). On day 6, the progression media was replaced with maintenance media (DMEM supplemented with 10% fetal calf serum). From days 0 to 6, the cells were exposed to the chemical reagents (DMSO, bilberry extracts 100 *μ*g/ml and anthocyanidins 100 nM). The cells are incubated at 37°C with 5% CO_2 _throughout the experiments.

### Oil Red O staining

On day 7, 3T3-L1 cells were fixed in fresh 10% formalin (pH 7.4) and stained with Oil Red O dye (Sigma-Aldrich, Tokyo, Japan). The lipid the staining was quantified by extracting the dye with 100% isopropanol and measuring the absorbance at 500 nm.

### Quantitative real-time PCR

Total RNA was isolated from 3T3-L1 cells using ISOGEN reagent (Nippon Gene, Tokyo, Japan). Total RNA was quantified using NanoDrop (Thermo Scientific) and reverse transcribed to cDNA using M-MLV Reverse Transcriptase Kit (Invitrogen Life Science Technologies, Tokyo, Japan) following the manufacturer's specifications. The expression level of genes in 3T3-L1 cells treated with bilberry extracts or anthocyanidins was assesed by quantitative RT-PCR (Stratagene Mx 3005 Sequence Detection System, Agilent Technologies, Tokyo, Japan). PCR amplification was performed using a QuantiTect SYBR Green PCR Kit (QIAGEN, Tokyo, Japan). The results are expressed as the fold increase relative to the controls after normalizing to the *β*-actin gene expression levels. The sequences of all primers used are shown in Table 1.

**Table 1:** Primer Sequences for quantitative PCR

Gene Name	**Accession Number (NCBI Nucleaotide)**	forward	Reverse
Pparγ	NM_011146.2	GTGCCAGTTTCGATCCGTAGA	GGCCAGCATCGTGTAGATGA
Adiponectin	NM_009605.4	GCACTGGCAAGTTCTACTGCAA	GTAGGTGAAGAGAACGGCCTTGT
C/ebpα	NM_007678.3	GTGTGCACGTCTATGCTAAACCA	GCCGTTAGTGAAGAGTCTCAGTTT
C/ebpβ	NM_009883.2	GTTTCGGGACTTGATGCAATC	AACAACCCCGCAGGAACAT
C/ebpδ	NM_007679.4	GATCTGCACGGCCTGTTGTA	CTCCACTGCCCACCTGTCA
Srebp1c	NM_011480.2	GTTACTCGAGCCTGCCTTCAGG	CAAGCTTTGGACCTGGGTGTG
Klf5	NM_009769.3	GGTCCAGACAAGATGTGAAATGG	TTTATGCTCTGAAATTATCGGAACTG
β-actin	NM_007393.2	AGCCTTCCTTCTTGGGTATGG	CACTTGCGGTGCACGATGGAG

### Western blotting

3T3-L1 preadipocytes were treated with DMSO, bilberry extracts or delphinidin and stimulated with insulin. After 5 min incubation, the cells were lysed in ice-cold RIPA buffer [10 mM Tris-HCl, 150 mM NaCl, 5 mM ethylenediaminetetraacetic acid (EDTA), 1% Triton X-100, 1% sodium deoxycholate, 0.1% sodium dodecyl sulfate (SDS), Complete mini and PhosSTOP (Roche Applied Science, Tokyo, Japan)]. An aliquot containing 10 *μ*g protein was separated by electrophoresis on a 7.5% SDS-polyacrylamide gel and bands were electrophoretically transferred to PVDF membranes (Millipore, Tokyo, Japan). The membranes were blocked for 1 hr at room temperature in 10 mM PBS containing 0.05% Tween 20 (PBS-T) and 5% BSA (Sigma-Aldrich, Tokyo, Japan). After washing with PBS-T, the membranes were incubated with target protein specific antibodies. All primary antibodies (IRS1, phosphorylated IRS1 (pIRS1), Santa Cruz, CA) were used at a dilution of 1:1,000. The secondary antibodies (horseradish peroxidase-conjugated donkey anti-rabbit IgG; (GE Healthcare, Tokyo, Japan) and horseradish peroxidase-conjugated rabbit anti-goat IgG; (Millipore, Tokyo, Japan) were used at a dilution of 1:10,000. The target proteins were detected using an Immobilon Western Chemiluminescent HRP Substrate (Millipore, Tokyo, Japan) according to the manufacturer's directions and detected with an LAS-3000 mini (Fujifilm, Tokyo, Japan). The band densities of Western blotting were quantified using Image Gauge version 4.22 program (Fujifilm, Tokyo, Japan).

### Microarray experiment and analysis

On day 2, the cells were harvested and total RNA was extracted. The microarrays were manufactured by Agilent Technologies (Santa Clara, CA). Total RNA (500 ng) was labeled and hybridized using a Whole Mouse Genome Microarray kit and Quick Amp Labeling Kit (Agilent Technologies) according to the manufacturer's protocol (Version 5.7). Hybridization signals were detected using a DNA microarray scanner G2505B (Agilent Technologies), and all scanned images were analyzed using Agilent feature extraction software (v9.5.3.1). Data were analyzed using GeneSpring GX 7.3.1 software (Agilent Technologies) and normalized as follows: (i) values below 0.01 were set to 0.01 and (ii) each measurement was divided by the 75th percentile of all measurements to compare the one-color expression profiles. The data presented in this manuscript have been deposited in NCBI's Gene Expression Omnibus and is accessible through GEO Series accession number GSE21157: http://www.ncbi.nlm.nih.gov/geo/query/acc.cgi?token=jvwfdguoakaucpk&acc=GSE21157. GSEA was used with HEAT http://hinv.jp/HEAT/search.php?lang=jp that has been described elsewhere [20].

## Results

### Bilberry extracts inhibited the adipocyte differentiation of 3T3-L1 cells

**Figure 1:**
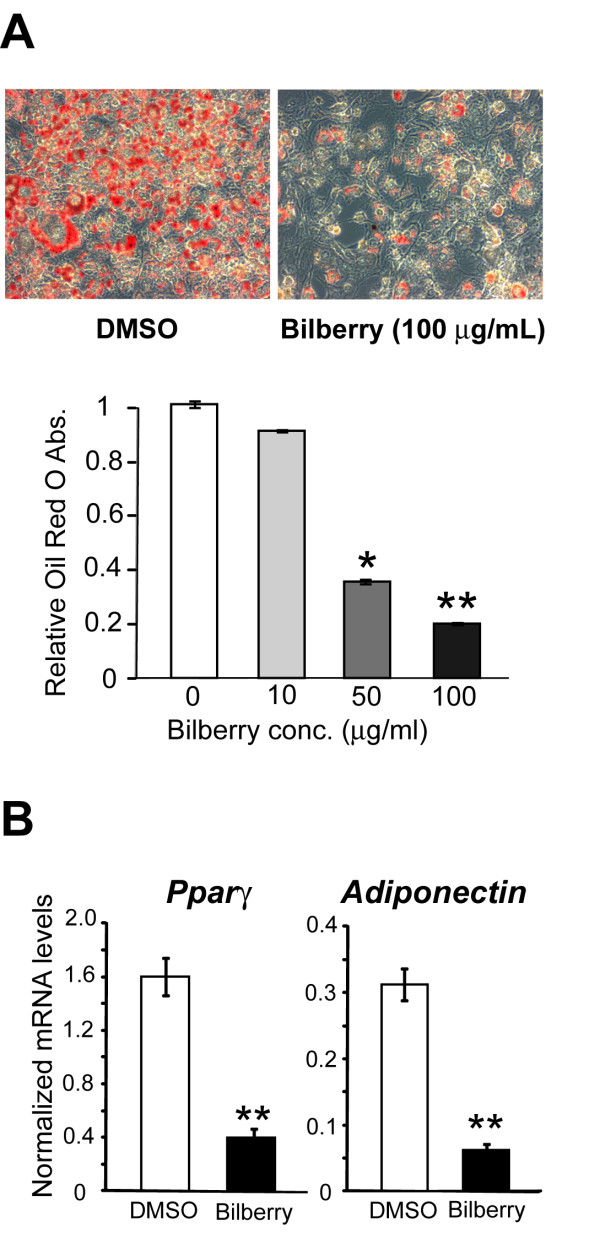
**Bilberry extracts inhibited 3T3-L1 adipocyte differentiation**. (A) 3T3-L1 preadipocytes were induced to differentiate in the presence of DMSO or 10, 50 or 100 *μ*g/mL of bilberry extracts for six days. On day 7, the cells were stained with Oil Red O and photographed. The lipid content in these cells was quantitatively determined as described in the Methods and Procedures. Data are expressed as the ratio of samples/control (DMSO treated cells) levels (n = 3). The results are shown as the mean ± S.D. (* *p *< 0.05, ** *p *< 0.001, Welch's t-test). (B) 3T3-L1 preadipocytes were induced to differentiate in the presence of DMSO or 100 *μ*g/mL of bilberry extracts. The Ppar *γ *and adiponectin mRNA levels on day 7 of differentiation were determined by the real-time RT-PCR (n = 3). *β *-actin was used as the reference gene (** *p *< 0.001, Welch's t-test).

First, we examined the effects of bilberry extracts on the differentiation of 3T3-L1 preadipocytes into adipocytes. Differentiation was initiated by treating the cells with or without bilberry extracts (0, 10, 50, 100 μg/mL). On day 7, we found that bilberry extracts markedly diminished the lipid accumulation in 3T3-L1 cells. Furthermore, bilberry extracts inhibited the differentiation of 3T3-L1 cells in a dose-dependent manner (Figure 1A). On the other hand, bilberry extracts had no effect on cell proliferation (data not shown). In addition, bilberry extracts down-regulated the mRNA levels of Ppar *γ*; a master regulator of adipocyte differentiation. Bilberry extracts had similar effects on the expression of adiponectin, which is a major adipocyte marker (Figure 1B). These results showed that bilberry extracts are capable of inhibiting adipocyte differentiation and diminished lipid accumulation with concomitant down-regulation of Ppar *γ*.

### Bilberry extracts inhibited the expression of adipocyte differentiation-associated transcription factors

**Figure 2:**
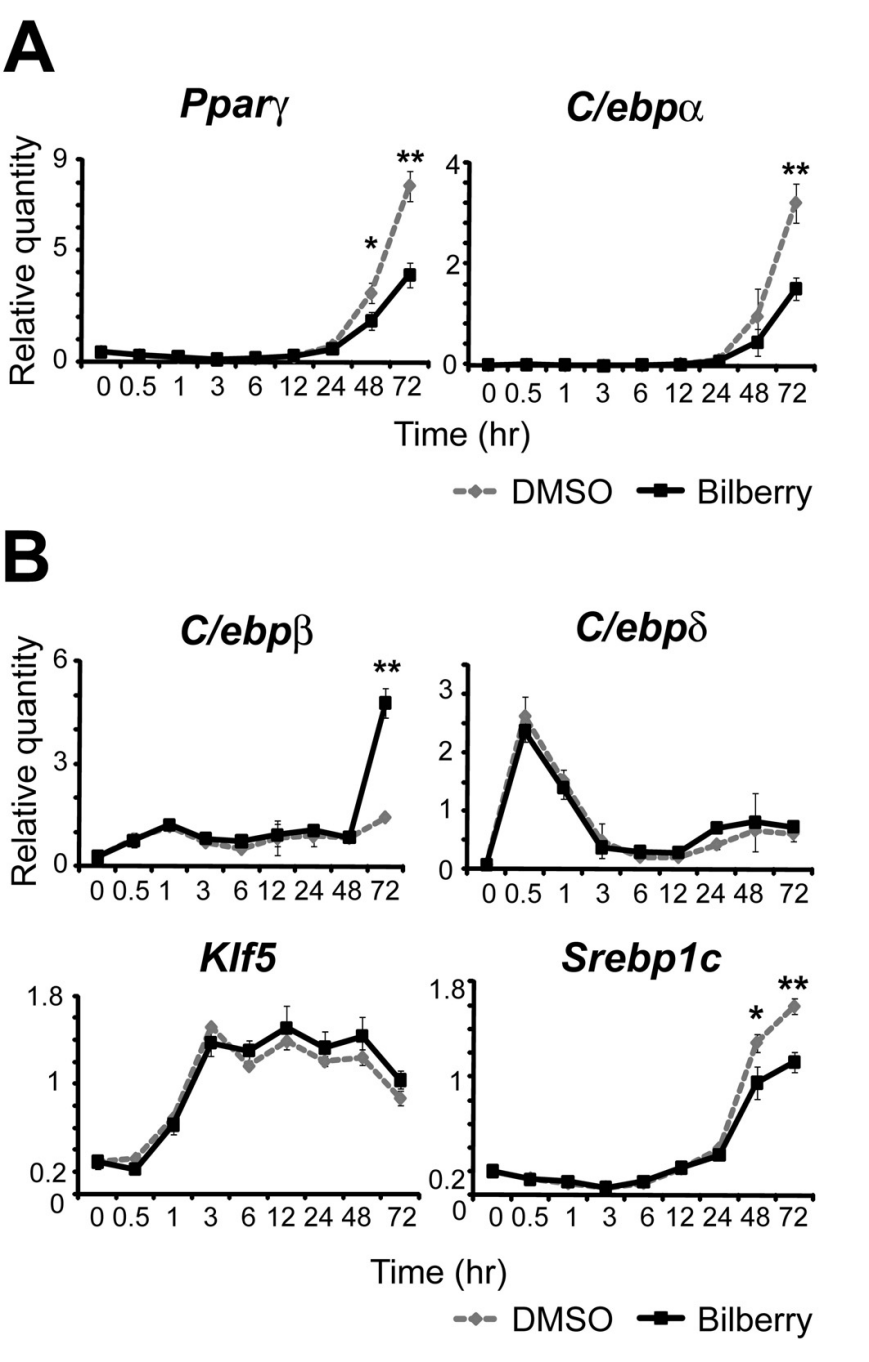
**Effects of bilberry extracts on the expression of adipogenesis-related transcription factors**. 3T3-L1 preadipocytes were incubated with DMSO or 100 *μ*g/mL of bilberry extracts and induced to differentiate. The expression levels of (A) master regulators of adipocyte differentiation; Ppar *γ *, C/ebp *α *, (B) upstream genes; C/ebp *β *, C/ebp *δ *, Klf5 and Srebp1c at various time points after induction were measured by real-time RT-PCR (n = 3). The results are expressed as the fold increase relative to the controls after normalizing to the *β *-actin expression levels. The results are shown as the mean ± S.D. (* *p *< 0.05, ** *p *< 0.001, Welch's t-test).

It has already been reported that transcriptional network of adipocyte differentiation is dominated by three pathways (insulin, glucocorticoid and cAMP). We investigated the effects of the bilberry extracts on the expression of transcription factors that are associated with these three pathways. As shown in Figure 2A, treating cells with bilberry extracts decreased the mRNA levels of Ppar *γ *and C/ebp *α *which play key-roles in adipocyte differentiation. We also evaluated the effects on C/ebp *β *, C/ebp *δ *, Klf5, and Srebp1c transcription factors that are upstream of Ppar *γ *and C/ebp *α*. Among these transcription factors, Srebp1c expression was significantly decreased on days 2 and 3, while the expression of C/ebp *δ *and Klf5 did not differ between the bilberry-treated and control cells (Figure 2B). These results raised the possibility that the main effects might be dependent on insulin pathway.

### The effects of anthocyanidins on adipocyte differentiation

**Figure 3:**
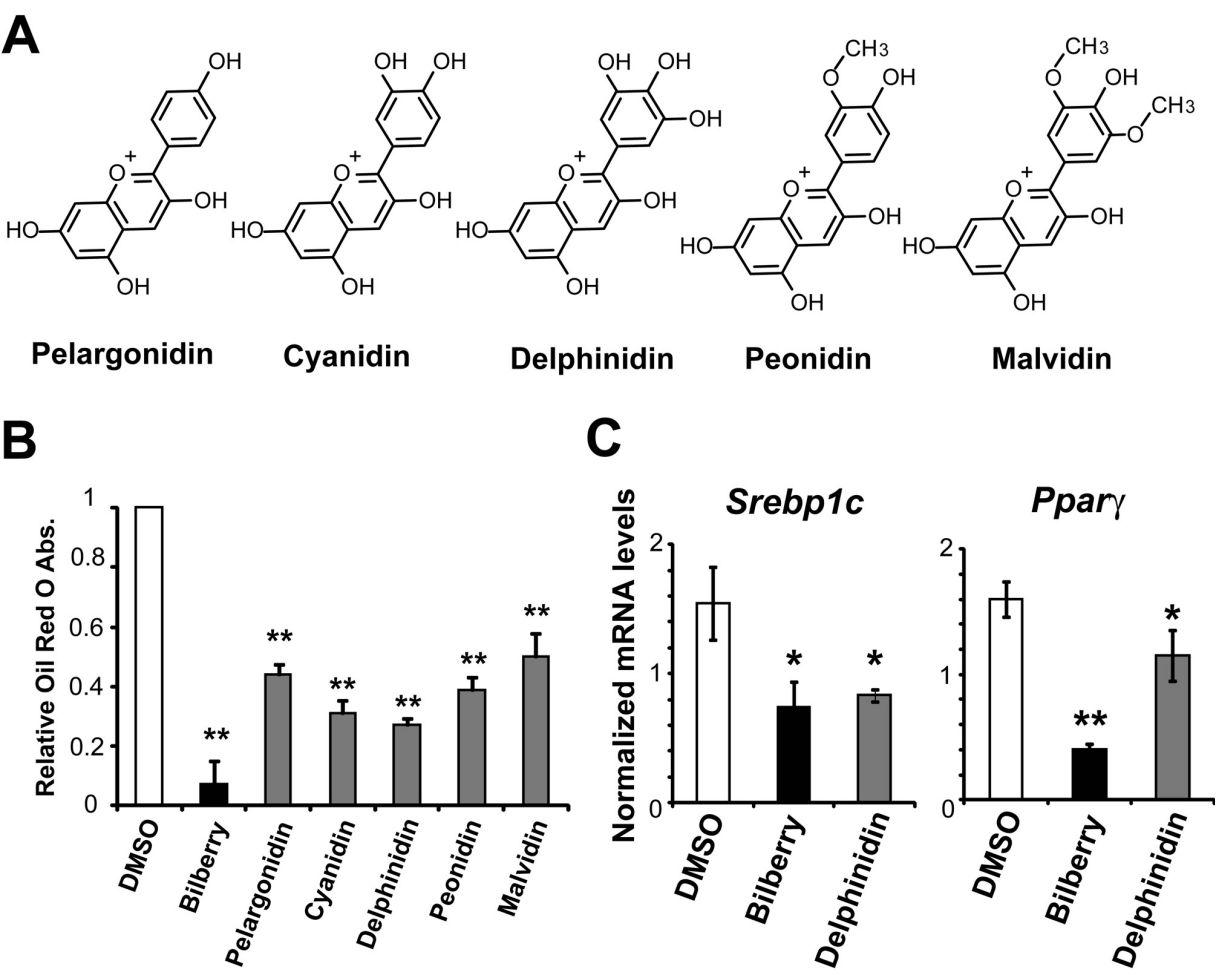
**Anthocyanidins contained bilberry extracts that inhibited 3T3-L1 adipocyte differentiation**. (A) Chemical structures of the five major anthocyanidins: pelargonidin, cyanidin, delphinidin, peonidin, and malvidin. (B) 3T3-L1 preadipocytes were induced to differentiate in the presence of DMSO, 100 *μ*g/mL of bilberry or 100 nM of the five anthocyanidins. On day 7, the cells were stained with Oil Red O and the lipid content in these cells was quantitatively determined (n = 3). Results are shown as the mean ± S.D. of the ratio of samples/control (* *p *< 0.05, ** *p *< 0.001, Welch's t-test). (C) 3T3-L1 preadipocytes were induced to differentiate in the presence of DMSO or 100 nM of delphinidin. The Srebp1c (day 2) and Ppar *γ *(day 7) mRNA levels were determined by the real-time RT-PCR (n = 3). *β *-actin was used as the reference gene (** *p *< 0.01, Welch's t-test).

As previously noted, bilberry is a rich source of anthocyanidins; a kind of flavonoids. To investigate whether the effect of bilberry extracts are derived from anthocyanidins, we examined the inhibitory effects of five major anthocyanidins [pelargonidin, cyanidin, delphinidin, peonidin, and malvidin (Figure 3A)] on differentiation of 3T3-L1 cells. These anthocyanidins occur naturally in bilberry at high concentrations [21]. Oil Red O staining revealed that all of the anthocyanidins also effectively inhibited lipid accumulation in 3T3-L1 cells (Figure 3B). In addition, out of the five anthocyanidin tested, delphinidin was most effective in down-regulating Ppar *γ *and Srebp1c mRNA levels (Figure 3C).

### Anthocyanidins and bilberry extracts inhibited insulin signaling pathway

**Figure 4:**
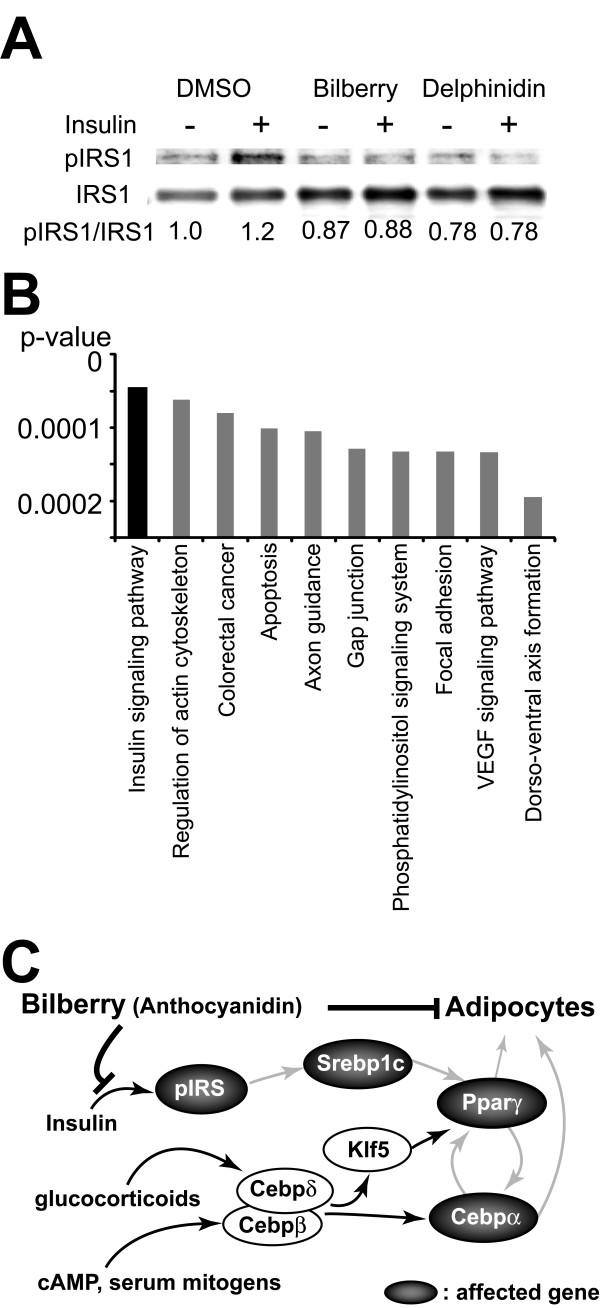
**Bilberry and anthocyanidins inhibit the insulin signaling pathway**. (A) 3T3-L1 preadipocytes were treated with DMSO, bilberry extracts (100 *μ*g/mL) or delphynidin (100 nM), and then stimulated with or without insulin (100 nM) for 5 min. Protein levels of IRS1 and pIRS1 were analyzed by western blot analysis. Band densities were quantified using Image Gauge 4.22 image analysis software. Protein levels are expressed as fraction of control (DMSO treated and without insulin stimulation cells) level. (B) Top significant pathways of the genes commonly altered in the "Bilberry", "Delphinidin" and "Cyanidin" by GSEA (Fisher's exact test). (C) The hypothetical scheme of a mechanism that anthocyanidins-enriched bilberry extracts inhibit adipocyte differentiation via insulin signal.

The altered expression of transcriptional factors led us to speculate about its potential impairing effects on the insulin signaling pathway, since these factors are known to play vital roles in the insulin signaling. We examined the effects of delphinidin and bilberry extracts on phosphorylation of IRS1 by insulin stimuli. 3T3-L1 preadipocytes were treated with 100 *μ*g/mL bilberry extracts or 100 nM delphinidin with 100 nM insulin for 5 min. When delphinidin or bilberry extracts were added in culture media, the ratio of pIRS1/IRS1 was not increased by insulin treatment as compared with the DMSO control (Figure 4A). Moreover, we compared the whole-genome expression profiles of adipocytes on day 2 (early stage of differentiation) under four different conditions (DMSO, bilberry, delphinidin, or cyanidin) by microarray analyses. The microarray analysis showed that 784 probes were commonly differentially expressed (fold change > 2) among the three conditions compared to the DMSO-treated cells. A subsequent GSEA analysis of these 784 probes revealed that altered-genes were associated with the insulin signaling pathway (KEGG: 4910, http://www.genome.jp/dbget-bin/get_pathway?org_name=hsa&mapno=04910 and were most significantly (p < 0.0001) overlapped with them in KEGG pathways (Figure 4B). These data indicate that anthocyanidin and bilberry blocked insulin signal during adipocyte differentiation (Figure 4C).

## Discussion

In this study, we examined the effect of bilberry extracts containing large amount of flavonoids, on adipocyte differentiation and the transcriptional network. The data showed that bilberry extracts inhibited the adipocyte differentiation of 3T3-L1 cells in a dose-dependent manner and also bilberry extracts down-regulated the expression of three transcriptional factors; Ppar *γ *, C/ebp *α *and Srebp1c. Next, we tested whether anthocyanidins; the major flavonoids of bilberry, had effects on adipocyte differentiation in a similar way. Indeed, anthocyanidin down-regulated the expression of Ppar *γ *and Srebp1c genes. Therefore, in presence of isulin stimuli, the level of Srebp1c is elevated which in turn results in up-regulation of Ppar *γ *and C/ebp*α *gene expression (Figure 4C). These results made us to hypothesize that the major effects of anthocyanidins was to block insulin signal. Western blotting analysis showed that bilberry extracts and anthocyanidin decreased the tyrosine phosphorylation of IRS1. In addition, genome-wide microarray experiments and GSEA revealed that in the early differentiation stages, bilberry extracts or anthocyanidins altered expression of genes in the insulin pathway. These data indicate that 1) bilberry extracts inhibit adipocyte differentiation via the insulin pathway and 2) these effects are primarily mediated by the action of anthocyanidins.

Recent investigations suggested that Srebp1c is the earliest transcription factors involved in adipocyte differentiation and Srebp1c is required to induce Ppar *γ *downstream of C/ebp *β *and C/ebp *δ *[22], these results suggest that bilberry extracts-induced inhibition of adipocyte differentiation may be predominantly associated with unchanged Srebp1c expression, accompanied by a strong inhibition of Ppar *γ *and C/ebp *α *in the transcriptional network. Notably, Kim et al. [23] demonstrated that ectopic expression of a dominant-negative Srebp1c inhibited preadipocyte differentiation, while overexpressing this protein significantly enhanced the adipogenic activity of Ppar *γ*. In their subsequent study, it was reported that insulin stimuli significantly increases Srebp1c expression in adipocytes [19].

More recently, Takikawa et al. [21] demonstrated that dietary bilberry extracts activate AMP-activated protein kinase (AMPK) in white adipose tissue. It was also reported that insulin-mediated activation of Akt can negatively regulate AMPK activity [24]. Therefore, it is deliberated that the inhibitory effects of anthocyanidin and bilberry extracts on IRS1 phosphorylation lead to Akt inactivation and AMPK activation. Additionally, Hafeez et al. [25] revealed that anthocyanidin; delphinidin induced apoptosis of prostate cancer cells and Lamy et al. [26] showed delphinidin inhibited phosphorylation of vascular endothelial growth factor (VEGF) receptor-2 in human umbilical vascular endothelial cells. Our GSEA results indicated that anthocyanidins altered genes known to be linked with "Apoptosis" and "VEGF signaling pathway" (Figure 4C) and these findings are consistent with other published reports. In essence of the data presented here and previous reports, it seems plausible that bilberry extracts or anthocyanidins might be interacting with the cellular receptors like IR or VEGFR. However, further investigation is required to validate this hypothesis.

To further elucidate the properties of bilberry extracts and anthocyanidins, we tested the inhibitory effects of five major anthocyanidins that are present in bilberry, including pelargonidin, cyanidin, delphinidin, peonidin, and malvidin, on 3T3-L1 differentiation. Although, all the five anthocyanidins effectively inhibited the lipid accumulation in 3T3-L1 cells, but the reasons for exhibiting differences in their inhibitory efficiencies was unclear. Perhaps, it may be from the number of hydroxyl groups present on the B-ring and the hydrophilic properties as shown in Figure 3A. The differences in their efficiencies displayed by these anthocyanidins warrants a more elaborate study to further elucidate the underlying mechanisms involved in their mode of action.

## Conclusions

We demonstrated that anthocyanidins-enriched bilberry extracts inhibited adipocyte differentiation via insulin pathway. These findings provide important insight and implications for preventing and treating obesity associated with metabolic syndrome.

## Competing interests

The authors declare that they have no competing interests.

## Authors' contributions

RS and MTan contributed to design the study, analysis and interpretation of data, and drafting of the manuscript. MTak, AH and BY assisted with interpretation of the result and critical revision of the manuscript. HT and MK supervised the analyses and helped to draft the manuscript. All authors have given their final approval of the submitted version of the manuscript.
